# Drug Discovery by Molecular Imaging and Monitoring Therapy Response in Lymphoma

**DOI:** 10.3390/ijms18081639

**Published:** 2017-07-27

**Authors:** Senthilkumar Kalimuthu, Ju Hye Jeong, Ji Min Oh, Byeong-Cheol Ahn

**Affiliations:** Department of Nuclear Medicine, Kyungpook National University School of Medicine and Hospital, 50, Samduk-dong 2-ga, Jung Gu, Daegu 700-721, Korea; senthilbhus@gmail.com (S.K.); zzu--@hanmail.net (J.H.J.); ojm0366@naver.com (J.M.O.)

**Keywords:** lymphoma, molecular imaging, drugs, PET-CT, bioluminescence

## Abstract

Molecular imaging allows a noninvasive assessment of biochemical and biological processes in living subjects. Treatment strategies for malignant lymphoma depend on histology and tumor stage. For the last two decades, molecular imaging has been the mainstay diagnostic test for the staging of malignant lymphoma and the assessment of response to treatment. This technology enhances our understanding of disease and drug activity during preclinical and clinical drug development. Here, we review molecular imaging applications in drug development, with an emphasis on oncology. Monitoring and assessing the efficacy of anti-cancer therapies in preclinical or clinical models are essential and the multimodal molecular imaging approach may represent a new stage for pharmacologic development in cancer. Monitoring the progress of lymphoma therapy with imaging modalities will help patients. Identifying and addressing key challenges is essential for successful integration of molecular imaging into the drug development process. In this review, we highlight the general usefulness of molecular imaging in drug development and radionuclide-based reporter genes. Further, we discuss the different molecular imaging modalities for lymphoma therapy and their preclinical and clinical applications.

## 1. Introduction

**Figure 1:**
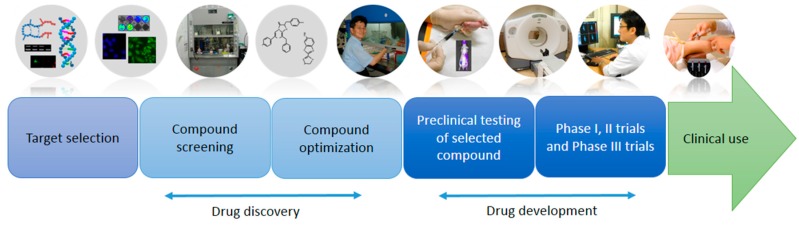
Processes in drug discovery and development. Although many drug candidates are evaluated in preclinical studies, very few compounds proceed to clinical trials and about one compound receives approval for administration and use in clinics. The selection of promising drug candidates in the early phase is critical for successful drug development. Molecular imaging contributes at various stages of the drug discovery and development processes.

The drug development process is a lengthy, high-risk, and costly endeavor. Although the specifics and duration of the process for drug development can be quite variable, in general, approval of a new drug from the beginning takes more than ten years [1]. Moreover, as a result of a dramatic increase in the required investment and a relatively constant rate of the introduction of novel drugs over the past 30 years, only few agents in the investigational new drugs (IND) category per year enter the market [1,2]. Fundamental research on disease pathophysiology is providing new drug targets and modifying agents that can inhibit or downregulate their function [3,4]. In 2013, only less than 1% of medicines among more than 5000 medicines in development were successfully approved by the Food and Drug Administration (FDA) [5]. In the selection and establishment of the target for diseases it is important to assess different drug discovery (Figure 1) approaches, so as to finally enable the clinical use of the drug. Molecular imaging has emerged as a new technology for both research and clinical drug development [3,6]. Hence, it is expected that an investment in molecular imaging technology will enhance drug development [7]. In drug development, before a drug enters a clinical development program, in particular when targeting chronic diseases with late clinical endpoints, it is important to identify reliable biomarkers that allow the validation of the drug’s mechanism of action on humans and the monitoring of drug efficacy [8]. Biomarkers may serve as alternatives for a clinical endpoint. Intensive research, including investigations at the level of gene transcription and translation, or metabolic alterations of the drug, is being conducted into delineating potential biomarkers for drug efficiency and safety [9].

Radionuclides used in the drug development process and in imaging modalities can themselves act as drugs by the cytotoxic effect of radiation emitted from the radionuclides [10]. The most remarkable characteristics of therapeutic β- or α-emitter radiopharmaceuticals comprise protection of the surrounding tissue from damage due to their short emission range. Radioimmunetherapy (RIT) is a targeted radionuclide therapy using a monoclonal antibody (mAb) tagged with therapeutic radionuclides to damage the target lesion [11]. Some radiopharmaceuticals are well established therapeutic options for certain diseases (such as thyroid cancers, osseous metastasis of prostate cancer) and have been successfully applied in thyroid clinics for more than 70 years, however, RIT is a relatively new therapeutic technology and only a few approved radioimmunotherapeutics are available in the market.

Lymphoma is a type of cancer that originates in the lymph system and part of the immune system, and can be divided into two categories: Non-Hodgkin’s lymphoma (NHL) and Hodgkin’s disease (HD) cover about 3–6% of all malignancies, which in the Western world is around the fifth most common type of cancer [12,13]. NHL and many examples of HD are possibly curable with proper chemotherapy or radiotherapy, and recurrence also can be treated with second-line treatment strategies [14]. Treatment guidelines are needed and accurate staging and response assessment is essential for decision-making. In that respect, molecular imaging is one of the essential tools for early diagnosis, initial staging, risk stratification, therapy response monitoring, and tumor recurrence detection [15]. Molecular imaging research is also contributing to the understanding of lymphoma pathogenesis and is helping to extend more effective care to patients. In this article, we first review the basis of molecular imaging modalities and contributions to drug discovery and development. Radionuclide-based therapies and clinical applications of the imaging modalities for lymphoma are also discussed.

## 2. Molecular Imaging for Drug Discovery

Molecular imaging can be defined as a “noninvasive visualization, characterization, and quantification of molecular and biochemical events that occur at the cellular or subcellular level within intact living organisms”. This approach generally exploits specific molecular probes, as well as intrinsic tissue characteristics from the source of image contrast, and provides an insight for understanding integrative biology, disease characterization, earlier detection, and evaluation of treatment [16]. Furthermore, it offers the possibility of repetitive, uniform, noninvasive, and comparatively automated studies of living subjects by identical or alternating imaging assays with different time intervals, and attaching statistics in longitudinal studies, also essential to reduce the number of animals and experimental costs [1,16]. Classically, when imaging for in vivo methods depends on gross anatomy it is referred as conventional imaging. With the introduction of imaging agents, it became possible to image physiological parameters in living subjects a called as functional imaging [17]. Molecular imaging, best example of function imaging, can be used to visualize drug targets and to monitor kinetics of administered drugs. For the newer molecular imaging tools to be useful, they have to possess high sensitivity, high spatio-temporal resolution. Still, limited target-specific molecular probes are available, so, more target-specific molecular probes must be developed to enhance values of molecular imaging for drug discovery and development [17,18]. Characteristics of molecular imaging modalities is shown in Table 1.

The advantage of molecular imaging techniques over conventional anatomical imaging is to study the biological properties in vivo with enough temporal and spatial resolutions without invasiveness. Various diagnostic imaging techniques are routine in clinical radiology, and they have an equivalent importance in the experimental research setting as well. Prior to clinical application, the temporal and spatial biodistribution of imaging probes or drugs and their diagnostic and therapeutic efficiencies should be assessed. Molecular imaging provides this data in preclinical settings, especially in animal models (in vivo). Increased availability of genetically engineered laboratory mice allows for better simulation of the clinical conditions. It is also important that the imaging techniques used to have high spatial resolution (10–100 µm to mm) and high sensitivity (millimolar to nanomolar) for small animals [1].

**Figure 2:**
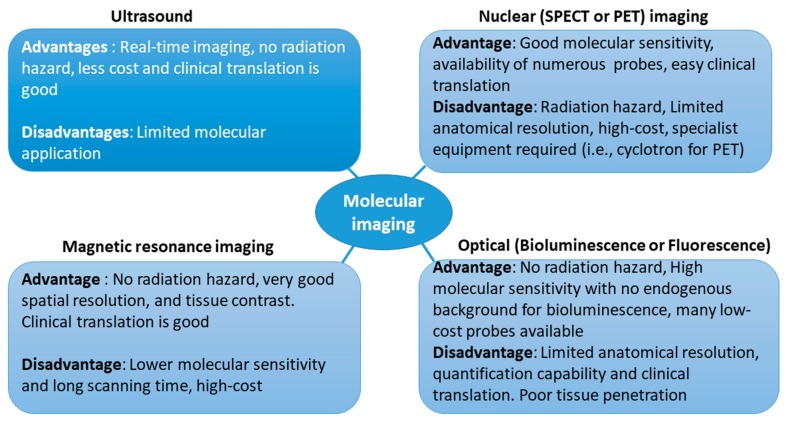
Advantages and disadvantages of different molecular imaging modalities. Positron emission tomography (PET), positron emission tomography; single-photon emission computed tomography (SPECT), single-photon emission computed tomography.

Each imaging modality (optical, nuclear, ultrasound, and magnetic resonance imaging (MRI)) has its own advantages and disadvantages (Figure 2). The important characteristics of each molecular imaging modality are listed in Table 1. Molecular imaging can be achieved with imaging technologies, such as optical imaging, nuclear imaging (e.g., single-photon emission computed tomography (SPECT); positron emission tomography (PET), MRI, and ultrasound imaging techniques, and it plays an important role for the “bench-to-bedside” translational approach [4,16,19,20].

Imaging technologies use the interaction of different forms of energy in tissues to image the body noninvasively. MRI and computed tomography (CT) depend solely on energy-tissue interactions, whereas others, such as PET or optical imaging, require the administration of radionuclides or optical probes. Imaging modality can be chosen mainly based on questions to be addressed for drug development, and the application of multimodal imaging might be good option because different imaging techniques are, in general, complementary rather than competitive. MRI has widely being used in pharmaceutical researches owing to its excellent soft tissue contrast properties. In addition, it yields valuable physiological information, even if very limited compared to nuclear or optical imagings. CT is the classical anatomical imaging method and is well suited for morphology-based studies. Nuclear imaging techniques, SPECT and PET, offer very high sensitivity required to evaluate drug distribution and pharmacokinetics, and to image specific molecular events. Depending on the ligands and radionuclides used, a myriad of molecular process can potentially be assessed.

Newer optical imaging techniques, such as fluorescence and Bioluminescence imaging (BLI), are of particular value for mapping specific molecular events and noninvasively tracking cells in living mice. They are also cheap, fast, and do not require radionuclides. A number of noninvasive technologies have been developed and actively used for clinical purposes, but have recently been miniatured to allow imaging of small animal with high resolution, which can be used to evaluate novel therapeutics in small animal models. Some imaging modalities fulfill the “bench-to-bedside” model, and can be applied in mice, other rodents, primates, and ultimately used in clinical trials. [3,17]. Among the different molecular imaging techniques, optical imaging based on bioluminescence and fluorescence has the highest sensitivity. In addition to the benefit of the exceptionally high signal to noise levels, optical molecular imaging provides multiplex imaging employing various probes having different optical spectrum, and needs the lowest cost for the instrument installation [16,21,22]. These benefits render optical imaging as the most prevalent technique for preclinical studies. An important drawback of this technique, however, is the absorption and scattering of light signals on their route to the detector system, making the visualization of the inner organs of an animal difficult and precluding further clinical applications [23]. To overcome these limitations, efforts have been made to generate reporters that emit photons at a longer wavelength, for example red light, which is transmitted through tissues more efficiently [21,23].

Furthermore, a quantitative three-dimensional (3D) image of optical imaging signals provides more accurate biological information compared with its planar counterpart. The 3D image is generated based on advanced mathematical algorithms that resolve photon scattering deep within tissue and localize the position of the source [24,25]. Nuclear imaging techniques, such as PET and SPECT (with nanomolar blood concentrations of injected radiotracers), provide the required 3D distribution of the administered tracer and possess high sensitivity and resolution with good tissue penetration depth. They have the potential to detect molecular and cellular changes that accompany diseases [23,26]. These advantages permit clinical and experimental applications of these imaging techniques. The assessment of treatment response is possible with quantitative nuclear imaging, and metabolic rates of diseased and normal organs can be measured with kinetic modeling [6].

MRI is a technique which uses a magnetic field and radio waves to generate detailed images of the organs and tissues within a body. MRI simultaneously provides molecular and anatomical information without a radiation hazard [16,27]. Diffusion-weighted imaging (DWI) exploits the variability of Brownian motion and provides information about the diffusion of water molecules in tissues. The main application of perfusion-weighted imaging comprises the evaluation of ischemic conditions and also measurements of cerebral hemodynamics at the microcirculation level. Magnetic resonance (MR) spectroscopy provides a defined spectrum that allows tissues to be interrogated for the presence and concentration of various metabolites, such as creatine, *N*-acetylaspartate, and choline [28]. These functional MR techniques have provided much information on physiological, biological, and metabolic structures, and high resolution anatomical information [28,29]. However, despite high tissue contrast and anatomical resolution, MRI is several orders of magnitude less sensitive than optical or nuclear imaging in obtaining molecular information [29]. This lower sensitivity requires the concentrations of the lesion-targeting molecular probes to exceed tracer levels. More effort needs to be directed toward the development of strategies to improve the sensitivity of MRI, such as dedicated coils, higher magnetic field strength, conditional MRI contrast agents, or activatable probes [4,7,30,31].

The molecular imaging approach could provide pharmaceutical research with solutions with high specificity, high sensitivity, and high temporal and spatial resolution. However, each molecular imaging technology has unique strengths and limitations, and it is not possible for a single modality to be ideal for all the possible applications [31]. Multimodality imaging tools combine technologies, such as CT, PET, and MRI, which have been emerging to overcome the drawbacks of single modality imaging, especially in vivo [22,23]. PET/MR and hybrid PET/CT were introduced in the late 1990s, and the success of the latter precludes the use of the PET system on its own [26]. The hybrid PET/MR approach rectifies the weaknesses of standalone PET and MRI, and is applied in clinical practice and preclinical research, having the advantage of no additional radiation and high tissue contrast compared to the combined PET/CT method [32,33]. Conventional MRI or functional MR spectroscopy can be incorporated into hybrid technologies and, thus, PET/MR is expected to play an increasing role in and make huge impact on translational research and preclinical drug discovery and development [34]. Furthermore, further developments concerning combined whole-body PET/MR scanning are underway in clinics [26,34].

## 3. Radionuclide-Based Molecular Imaging

The success of radionuclide imaging for the discovery and development of new drugs, either measured in in vitro samples or detected externally from a tissue sample or a patient, relies on the use of adequate radionuclides [35]. The development of powerful radiopharmaceuticals requires careful consideration of radionuclide selection. Γ- or positron-emitting radionuclides can be used for labeling of diagnostic radiopharmaceuticals, while, in contrast β- or α-emitting radionuclides will be better for therapeutic radiopharmaceuticals. The decay properties of the attached radionuclide need to be balanced with in vivo targeting and clearance of the carrier molecule [36]. Selection of radiolabeling techniques should be determined by the structure of the probe molecules, and biological characteristics of the molecules must be maintained after the labeling.

Noninvasive molecular imaging with reporter genes in the field of biomedical imaging which holds abundant promises for therapy response and also diagnosis. Reporter gene-based imaging comprises one type of “molecular imaging”, a recently-coined term that is used to describe visualization of normal and abnormal processes at a cellular or molecular/genetic level, in both space and time [37]. Genes selected as reporters either confer easily identifiable and measurable characteristics onto the cells expressing them, or are selectable markers [38,39]. In the classical biological approach, high numbers of experimental animals are required to conduct in vivo experiments and tissue samples are needed to monitor time point changes. In the noninvasive imaging reporter gene technology, the transgene expression reading obtained at each time point from the same subject provides detailed information. Certain reporter genes encode a protein that could be a therapeutic target, which can be indirectly visualized by trapping their imaging probes. There are a number of approaches for reporter gene imaging, including optical, radionuclide and MR imagings [40,41,42,43]. Radionuclide-based reporter genes are generally categorized into three groups based on the interaction between reporter proteins and their respective probes; these are enzyme, receptor genes, or transporter [39]. First, a reporter gene that encodes an enzyme that is capable of trapping a specific tracer by action (for example, phosphorylation) of the enzyme; Second, a reporter gene that encodes for an intracellular and/or extracellular receptor, which is capable of binding a specific tracer; Lastly, a reporter gene that encodes a transmembrane transporter, which is capable of transporting a specific tracer into the cells. A main disadvantage of the reporter gene system is that a reporter gene first needs to be introduced into the cells under consideration through delivery vectors (for example, viral vectors or liposomes) [1].

The imaging reporter gene enables noninvasive assessment of transgene expression in in vitro and in vivo studies. Radionuclide-based reporter gene imaging has excellent sensitivity, high resolution, and extremely good tissue penetration depth [39], and the imaging can be achieved with different imaging modalities, such as MR, optical, radionuclide, and ultrasound-based techniques, X-ray, and so on [44,45,46,47]. So far, the radionuclide-based reporter gene method is currently the only clinically available imaging modality for transgene expression [48].

The radionuclide-based reporter gene system is useful for diagnostic and/or therapeutic applications and the appropriate radionuclides can be selected for various purposes. γ ray emitting radionuclides are used for the visualization of reporter gene-expressing cells. β ray emitting radionuclides killing reporter gene-expressing cells. Visualization of gene expression and cell killing can be performed simultaneously or sequentially. Over the past decade, various enzyme/prodrug systems, such as yeast cytosine deaminase/5-fluorocytosine (yCD/5-FC), thymidine kinase/ganciclovir (TK/GCV), and nitroreductase/CB1954 (NTR/CB1954), have been used for suicide gene therapy in cancer treatment. CD [49,50,51], herpes simplex virus type 1 thymidine kinase (HSV1-TK) [51,52], NTR [53], and NTR mutant deoxycytidine kinase (dCK) [54] can convert a prodrug into a cytotoxic drug; thus, a therapeutic radionuclide becomes obsolete in therapy. Receptor-based monitoring of somatostatin receptor type 2 [55], bombesin receptor [56] and transported based monitoring of sodium iodide symporter [57], norepinephrine [58], can be used for molecular imaging. Furthermore, one of the first noninvasive reporter gene imaging applications was based on HSV1-TK and described in 1995 [59,60]. HSV1-TK reporter system is a radiotracer enzymatic assay similar to the FDG (fluorodeoxyglucose)–hexokinase system that is widely used for glucose utilization imaging [60]. Owing to their theranostic usability, radionuclide-based reporter genes have been widely applied in cell-based therapy in preclinical studies [39]. However, two important issues need to be resolved prior to clinical translation of theranostic applications. First, optimization of the cell-based therapy and, second, visualization of the administered cells in vivo. Therapeutic cells tagged with a radionuclide-based reporter gene can be detected by nuclear molecular imaging owing to the excellent tissue penetration capability of γ rays emitted from the appropriate probe labeled with radionuclide. The administered cells can thus be monitored by reporter gene imaging in a cell-based therapy. The important issue here, however, is safety, because uncontrolled proliferation of the administered cells can occur, specifically in the case of embryonic stem cell therapy [61]. Introduction of a suicidal gene into therapeutic cells is an example of a safety back-up strategy [62]. Moreover, therapeutic cell visualization and removal using radionuclide-based imaging reporter gene in a mouse model have worked well to resolve the above-mentioned concerns [63].

## 4. Development of Therapeutic Strategies with Optical Imaging in Preclinical Models

Preclinical mouse models are important and helpful tools for studying biology and disease pathophysiology, and for developing therapeutic strategies for certain diseases. Detailed preclinical evaluation is needed before novel therapeutic approaches can undergo translation into clinical trials [64]. Advances in fluorescent probe design and optical detection technology facilitate application of optical imaging technologies for drug discovery and development. Biologically compatible near-infrared (NIR) probes can be visualized safely even in vivo animal models, and the highly sensitive photon-detection technologies provide better imaging results even with fluorophores having low photon yield.

Bioluminescence and fluorescence imagings became popular in the field of drug discovery and development due to their high sensitivity, low cost, versatility, and high-throughput capability. Fluorescent proteins allow actual images rather than photon-counting of luciferase imaging [65,66,67,68]; however, BLI represents the biochemical reaction of luciferases and their substrates. Unlike fluorescence techniques, bioluminescence techniques do not generate inherent background signal, which renders BLI more sensitive than fluorescence imaging [17,69].

**Figure 3:**
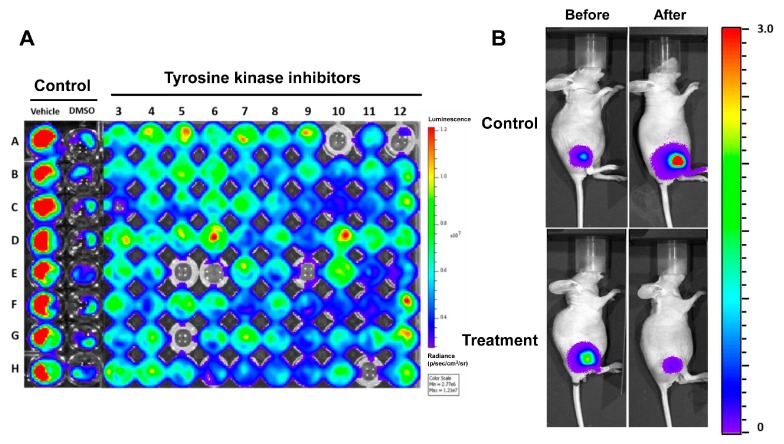
Drug screening and therapeutic drug monitoring by Bioluminescence imaging (BLI). (**A**) Drug screening by BLI. Stable luciferase (FLuc)-transfected cancer cells can be used for high throughput screening (HTS). FLuc activity of cancer cells treated with 5 µM tyrosine kinase inhibitors was analyzed by BLI imaging after 24 h. Compounds that decrease FLuc activity were selected and used further in target study and preclinical drug efficiency; (**B**) Therapeutic drug monitoring by BLI imaging. Stable FLuc-transfected cancer cell was injected into the subcutaneous tumor (Xenograft) and then therapeutic effect was monitored. Decrease in FLuc activity was observed in the drug-treated mouse after 2 weeks of treatment.

Optical imaging plays an important role in high-throughput in vitro chemical screening and is also a powerful and versatile imaging platform for in vivo pre-clinical animal studies. High-throughput screening (HTS) has widely been used to screen hit compounds from compound libraries in academia and the pharmaceutical industry, as a central paradigm of drug discovery and development. HTS of compound libraries against pharmacological targets is one of the key strategies in modern drug discovery [70]. Luciferase is best known as a genetic imaging reporter in HTS applications. Numerous cellular events with application to drug discovery are associated with the regulation of gene transcription [70]. HTS are frequently performed by means of miniaturized cell-based assays which enable chemical libraries to be screened for molecules that present different biological activities [71]. In BLI imaging modality, a luminescent protein or enzyme can be transfected into cells and used for drug screening and therapy response (Figure 3). The transfected cells are then implanted into an animal [72,73]. The light emitted from the implanted cells is then imaged and used to assess treatment response or progress of the diseases. This in vivo optical imaging modality also allows easy recognition of a molecular or biological process without animal sacrifice.

Research into molecular imaging is also contributing to our understanding of lymphomas and helping to direct more effective care of patients with certain types of the disease. Each year, new cases of lymphoma are diagnosed and more people die from the disease. An urgent need exists for the development of new diagnostic and therapeutic technologies for lymphoma, and molecular imaging can contribute to these developments. Adult T-cell lymphoma/leukemia (ATLL) is caused by human T-cell lymphotropic virus type 1 (HTLV-1). A bioluminescent mouse model has been developed to investigate new therapies for humoral hypercalcemia of malignancy and ATLL [74].

Rituxan (Rituximab), a chimeric immunoglobulin G1 (IgG1) monoclonal antibody (mAb) directed against the CD20 antigen, has an improved therapeutic effect in NHL. Dayde et al. showed that rituximab prevented the development of lymphoma tumor in mice treated with 6 mg/kg of rituximab 1 day after inoculation with EL4-huCD20-Luc cells, which was confirmed with BLI analysis [75]. Proteasome inhibitors, such as PS-341, suppress nuclear factor κB (NF-κB) activity by inhibiting the degradation of inhibitor κB (IκB) family members [76]. PS-341 has been used as a chemotherapeutic agent for lapsed multiple myeloma [77]. PS-341 and zoledronic acid, a bisphosphonate, were administered alone or in combination to treat mice xenografted with HTLV-1 infected cells that developed predominantly mesenteric lymph node lymphoma, five weeks after inoculation. BLI imaging showed significantly lower signal in mice treated with either PS-341, zoledronic acid, or their combination, compared with the empty vehicle control group [74].

Terziyska et al. isolated acute lymphoblastic leukemia (ALL) cells from ALL patients and transduced a membrane-bound form of Gaussia luciferase (GLuc). They performed imaging-guided preclinical treatment trials in a mouse model having the tranduced acute lymphoblastic leukemia cells and demonstrated that individual ALL samples retained their individual sensitivities towards conventional cytotoxic drugs [78]. Thus, GLuc-based in vivo imaging using an individualized preclinical model enables treatment trials at a new level of accuracy and precision. This patient derived ALL animal model is facilitating a detailed preclinical analysis of important therapies to prepare their translation into the clinic and might address the most demanding clinical questions, such as treatment failure and relapse [78].

To assess drug efficacy in a central nervous system (CNS) lymphoma xenograft model, Kadoch et al. transduced the luciferase gene into Raji cells using the lentivirus transfection method, thereby enabling cell visualization via in vivo BLI [79]. The authors investigated the response of intracranial luciferase-modified Raji xenografts to orally administered temozolomide (250 mg/kg/days for 5 days), an alkylating agent commonly used in primary CNS lymphoma therapy. The treatment group reproducibly revealed that significant delay in tumor progression, as shown by BLI, had delayed the onset of neurologic symptoms, and prolongation of survival compared with control mice. Whereas Raji cells were sensitive to temozolomide in a dose-dependent manner in vitro, Raji tumors rapidly exhibited resistance to this agent in vivo. This was demonstrated by in vivo BLI, when the overall survival of treated mice bearing CNS lymphoma xenografts did not exceed 23 days even when the temozolomide dose was increased to 300 mg/kg/days [79].

Near-infrared fluorescence (NIRF) imaging is developing revolutionary new technologies for the visualization of veins and also the detection and monitoring of brain injuries and malignant cancers. NIRF-based optical imaging is promising for a clinical diagnostic imaging for solid tumors by its high sensitivity [80]. The mAb based functional probe can be used for in vivo optical imaging of the lymphoma cells [81]. The noninvasive imaging can also help in the early detection of NHL, and to characterize the behavior of tumors [82].

## 5. Clinical Molecular Imaging for Lymphoma

The outcomes for lymphoma have significantly improved over the past few decades with 5-year survival rates, especially in NHL, increasing from 47% in 1975–1977 to 71% in 2003–2009 [13]. This dramatically improved survival is attributed to newer chemotherapeutic regimens and the inclusion of monoclonal anti-CD20 agents in combination strategies for NHL. In addition, tremendous advances in immunophenotyping, cell biology, and molecular genetics of lymphoma have led to newer risk stratification strategies, as well as the development of targeted agents [83]. With newer and more effective therapies for lymphomas, the need for accurate staging systems and standardized criteria for response becomes even more critical. Clinical trials exhibiting the efficacy of new drugs are essential before these new drugs are approved to enter the market. Multimodal molecular imaging modalities using a nuclear imaging technique, including PET/CT and PET/MR using glucose or amino acid tracers, are examples of standard imaging modalities for assessing the therapeutic response of lymphoma to new drug candidates. Therefore, nuclear molecular imaging, as the only clinically translated molecular imaging method, is a widely used assessment tool for the efficacy of new drug candidates in many clinical trials targeting lymphomas.

### 5.1. ^18^F-Fluorodeoxyglucose (FDG) PET/CT

Since the early 2000s, the assessment of lymphoma has been essentially based on clinical examination, CT, and bone marrow (BM) biopsy. Traditionally, imaging has played a fundamental role in the initial staging and surveillance of lymphoma, and the Cotswold classification was the first one to formally include CT scans [84]. Functional imaging with ^18^F-FDG PET/CT is widely used in the staging and evaluation of therapy response in lymphomas, overcoming the limitations of conventional anatomic imaging modalities. One advantage of ^18^F-FDG PET/CT scans over CT is that they enable discrimination of a viable tumor from scar and fibrosis in residual tumor mass. ^18^F-FDG PET/CT provides metabolic information on tumors based on the assumption that cancer cells are generally characterized by increased glucose utilization. ^18^F-FDG PET/CT was incorporated for response assessment in the International Harmonization Project recommendations published in 2007 [85].

**Figure 4:**
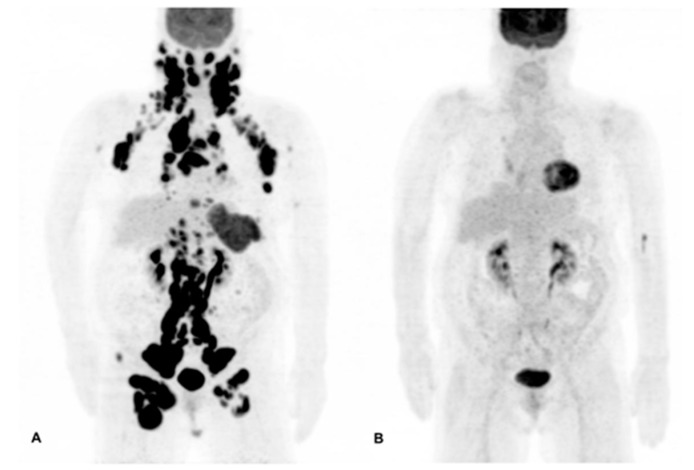
^18^F-Fluorodeoxyglucose *(FDG) PET*/CT imaging of the effect of R-CHOP therapy in a patient with follicular lymphoma. A 70-year-old female patient was diagnosed with follicular lymphoma after lymph node excision. The initial ^18^F-FDG PET/CT imaging (**A**) revealed hypermetabolic lesions in the palatine tonsils, spleen, and lymph nodes of the neck, axilla, mediastinum, paraaortic, iliac, and inguinal regions. After three cycles of R-CHOP therapy (rituximab, cyclophosphamide, doxorubicin, vincristine, and Prednisolone), a follow up imaging (**B**) revealed that the hypermetabolic lesions have disappeared, representing a complete response to therapy.

^18^F-FDG PET/CT is now an obligatory diagnostic procedure for initial staging and end-of-therapy treatment response assessment in FDG avid lymphomas. ^18^F-FDG PET/CT assessment has over 95% sensitivity and specificity. In 10 to 20% of cases, changes in staging can be made by ^18^F-FDG PET/CT, particularly in disease staged at I/II on CT, sometimes leading to changes in therapeutic management [86]. ^18^F-FDG PET/CT can even replace BM biopsy analysis for HL, and according to the new Lugano classification, BM biopsy can be distinguished in diffuse large B-cell lymphoma (DLBCL) with no evidence of BM involvement using PET/CT [87]. Based on the meta-analysis, specificity and sensitivity of ^18^F-FDG PET for residual disease detection and after first-line therapy for HL were 84% and 90%, respectively, 72% and 100%, respectively in aggressive NHL [88]. Furthermore the role of interim ^18^F-FDG PET/CT, performed after a few cycles of chemotherapy (Figure 4), is actively investigated in clinical trials for risk-adapted therapy strategies [89,90,91].

The international consensus for lymphoma, such as the International Harmonization Project and Lugano classification, involves standardized performance and interpretation of ^18^F-FDG PET/CT using a five-point visual scale. Such consensus guidelines of ^18^F-FDG PET/CT are helpful for accurate comparisons between studies, for accelerating standardization of uniform reporting system and for identifying optimal regimens in the clinical trials or clinical practices [87]. In addition, the quantitative parameters, including the metabolic tumor volume and total lesion glycolysis, which may better reflect the overall tumor burden [92], are now recognized as valuable tools to improve the robustness of therapeutic follow-ups.

### 5.2. Non-FDG PET/CT

With the introduction of new tracers, PET offers new potentially valuable parameters for lymphoma imaging.

#### 5.2.1. ^18^F-Fluorothymidine (^18^F-FLT)

3′-[^18^F] fluoro-3′-deoxythymidine (^18^F-FLT) is a structural analog of the DNA constituent thymidine and a representative marker of cellular proliferation. Because residual lymphoma would be expected to exhibit a high level of proliferation, whereas inflammation would not, ^18^F-FLT PET/CT might be suitable for distinguishing these two states, which cannot be discriminated by ^18^F-FDG PET/CT. Recent pilot study demonstrated that ^18^F-FLT PET/CT scan excellently differentiate residual lymphoma from post-treatment inflammatory changes in patients showing ^18^F-FDG avid lasting masses (90% sensitivity and 100% specificity for residual disease) [93]. Furthermore, early interim ^18^F-FLT PET/CT seems to be a significant predictor of progression-free survival and overall survival in patients with aggressive NHL [94], and a potential tool for predicting complete response in DLBCL patients with R-CHOP (rituximab, cyclophosphamide, doxorubicin, vincristine, prednisone) therapy [95].

#### 5.2.2. ^11^C-MET (Methionine)

^11^C-methionine (MET) is a radiolabeled amino acid used to monitor amino acid metabolism in tumors. MET uptake represents higher amino acid incorporation and protein synthesis, which is associated with cell proliferation. Cancers are, possibly universally, MET-dependent and require excess MET. Therefore, when many tested cancer cells are deprived of MET, a condition that is generally nontoxic to normal cells, they arrest development and eventually die [96]. ^11^C-MET has been widely used in detection of many cancers including lymphoma. Given that ^11^C-MET can cross the blood brain barrier, it is more useful in assessing the therapeutic response of brain tumors to radiotherapy than ^18^F-FDG [97]. Nuutinen et al. investigated whether ^11^C-MET uptake was associated with the histological grade of malignancy and survival in NHL and HL patients. The authors demonstrated that the technique was able to differentiate high-grade lymphoma from low-grade lymphoma if using the influx constant. However, prediction of survival was not feasible with ^11^C-MET PET/CT [98]. ^11^C-MET is useful for delineation of CNS lymphoma, because ^11^C-MET has lower uptake in the normal brain than ^18^F-FDG does, and for monitoring the therapeutic effect of irradiation [98]. Ogawa et al. performed ^11^C-MET PET in CNS lymphoma patients before and after radiation therapy, and demonstrated decreased tracer uptake after the therapy [99].

### 5.3. PET/MR (Magnetic Resonance)

An integrated whole-body PET/MR scanner has been recently introduced and is expected to potentially exceed the advantages of PET/CT because MR data provide high soft tissue contrast and can provide accurate anatomical details. Despite different scanner geometry and attenuation correction approaches, qualitative lesion detection is highly reproducible with hybrid PET/MR and PET/CT, and the standardized uptake values (SUV) values of PET positive lesions correlate well between the two modalities [100]. Platzek et al. evaluated the use of sequential PET/MR for lymphoma staging in 27 lymphoma patients and concluded that PET/MR is feasible for this application [101]. The MR component of a PET/MR scanner adds functional information derived from MR technologies and, especially in DWI, can offer information about tumor cellularity and improve tumor detection by direct comparison with metabolic information from the PET component. Lin et al revealed that result of DWI was 94% concordance with the findings of ^18^F-FDG PET/CT [102]. Furthermore, recent data indicated that the sensitivity (90%) of whole-body MRI equals that of ^18^F-FDG PET/CT for the detection of bone marrow involvement in HL but is less specific (75%) [103]. The benefit of MR in combination with ^18^F-FDG PET is not yet clear but DWI might play a complementary role for baseline imaging and assessment of treatment response in lymphoma.

### 5.4. Clinical Molecular Imaging for Experimental Therapies

Clinical trials using ^18^F-FDG PET/CT for the evaluation of many experimental therapies for lymphoma, including combinations of experimental drugs, are ongoing. For example, the combination of lenalidomide and R-CHOP, phase II trial, was evaluated for safety and efficacy by assessing the final response with ^18^F-FDG PET/CT in elderly patients with untreated DLBCL. Forty-five patients (92%) achieved a response (complete remission (CR), 86%; partial response (PR), 6%) [104]. Phase I study of a novel oral Janus kinase 2 (JAK2) inhibitor (SB15180) for patients with relapsed or refractory Hodgkin’s or NHL also included ^18^F-FDG PET/CT to evaluate baseline disease status and treatment response [105].

Furthermore, the clinical response of radioimmunotherapy (RIT) was demonstrated with ^18^F-FDG PET/CT in many studies because the clinical application of RIT has greatly expanded in recent years [106]. Early DLBCL patients in phase II studies were monitored with ^18^F-FDG PET/CT during experimental therapies of R-CHOP followed by anti-CD20 RIT. In the present study, 89% percent of patients achieved functional CR after R-CHOP plus RIT. At 5 years, 78% of patients remain in remission and 94% are alive [107]. In addition, an ongoing phase I/II trial of Tenarad RIT (^131^I-F16SIP) evaluated the treatment efficacy using ^18^F-FDG PET/CT in patients with refractory HD. Tenarad is a fully human mini-antibody, or small immunoprotein (SIP, 80 kDa), labeled with ^131^I, and targets the extra-domain A1 of tenascin-C, which is one of the best characterized markers of angiogenesis [108].

PET/CT imaging using a mAb labeled with a positron-emitting isotope, such as ^89^Zr, could be useful for visualizing the biodistribution of the individual radiotracer, subsequently RIT with ^90^Y-labeled rituximab in CD20+ B-cell lymphoma [109]. Janik et al. assessed the clinical response to ^90^Y-daclizumab (radiolabeled anti-CD25 antibody) therapy for HL by using SPECT/CT with ^111^In-daclizumab and ^18^F-FDG PET/CT [110]. ^111^In-daclizumab was administered to identify biodistribution and tumor targeting. SPECT imaging with ^111^In-daclizumab was congruent with ^18^F-FDG findings. In 46 evaluable HL patients treated with ^90^Y-daclizumab, there were 14 CRs and nine PRs.

Ibritumomab and Tositumomab are antibodies that target and bind to the CD20 antigen found on the surface of malignant B cells; therefore, labeling these antibodies with β-emitting radionuclides allows this radiation to kill the target cells along with others nearby. It mainly targets low-grade or follicular B-cell NHL and newly diagnosed follicular NHL following a response to initial anticancer therapy [106]. The mAbs Zevalin (^90^Y-ibritumomab tiuxetan) and Bexxar (^131^I-tositumomab) are notable representatives of FDA-approved drugs for the treatment of NHL. Unfortunately, Bexxar was withdrawn from the market in 2014 [10,11].

### 5.5. Personalized Medicine

With the shift of the medical paradigm into era of personalized medicine, the enormous needs are requested for tailored drugs based on individual response to each patient (Nuclear Medicine in the Era of Precision Medicine [111]). Clinical molecular imaging methods including PET are required to select appropriate patient group for certain drugs on the basis of imaging biomarker. In addition, radionuclide theranostics, one of the representatives for personalized medicine, uses pre-therapy low-dose diagnostic/theranostic imaging followed by higher-dose therapy in the same patient [112]. T-lymphocytes are one of key components of immune response which eliminating abnormal cells and infectious agents from the body. Adoptively transferred cytotoxic T-lymphocytes have been developed to control resistant cancers and in vivo monitoring of the administered T-lymphocytes are warranted to optimize the therapy and predict the therapeutic effect. Koehne et al. monitored radionuclide-based reporter gene transduced T cells noninvasively with PET imaging [113]. PET imaging allows for quantifying cell signals of the regions of anatomic interest. However, PET signal detection requires knowledge of cell numbers in different regions. Su et al. determined the correlation of PET signal with cell number and characterized the cellular limit of detection for PET imaging [114].

Antibody-drug conjugates (ADCs) are powerful biopharmaceutical drugs developed for targeted therapy against cancerous diseases. Arming of mAbs by connecting them with certain cytotoxic drugs enhances the targeting efficiency of the therapeutic agent, specifically to certain tumors, and results in a valuable enhancement of antitumor activity. Brentuximab vedotin (Adcetris) is an ADC directed to the protein CD30, which is expressed in classical HL and systemic anaplastic large cell lymphoma [115,116,117]. mAbs are among the most rapidly expanding classes of therapeutics for the treatment of cancer [118]. Rituximab is the only commercially available unmodified mAb that demonstrates antitumor activity in HL [119,120]. The US FDA has approved the use of brentuximab vedotin in the treatment of relapsed HL after failure of autologous stem cell transplantation or multi-agent chemotherapeutic regimens [117]. In addition, antitumor mAbs can be used as therapeutics after labeling with therapeutic radionuclides. The radiolabeled mAb conjugates are able to kill cancer cells at a distance of several cell diameters by the substantial range of emitting particles and, thereby, may also kill antigen-negative tumor cells adjacent to antigen-expressing cells [121,122,123]. Daclizumab (humanized anti-Tac, i.e., anti-CD25) can be armed with a β-particle emitter ^90^Y. Daclizumab targets the 55-kDa IL-2Rα (CD25) subunit that is constitutively expressed on Treg cells but not on other resting normal cells [124,125]. Lim et al. reported that patients with relapsed or refractory B-cell NHL were treated by RIT with radioiodinated human/murine chimeric anti-CD20 mAb rituximab (¹³¹I-rituximab). Contrast-enhanced ¹⁸F-FDG PET/CT scans before therapy and after one month allowed for the tumor sizes and maximum standardized uptake values (SUV_max_) to be measured [126]. Therefore, molecular imaging technology is more useful for new drug discovery and therapeutic monitoring.

Drugs and therapies for lymphoma are summarized in Table 2.

## 6. Conclusions

The unique features of molecular imaging allow us to expand our knowledge of the therapeutic targets for lymphomas and pathways involved in the initiation and progression of lymphomas, and provide bridges to clinical applications in diagnosis, staging, therapeutic target determination, and monitoring therapeutic response. Therefore, molecular imaging is exceedingly useful in drug discovery and development for lymphomas, by accelerating the entire process. With time, it has become crucial for the success of the development of new drugs. At present, molecular imaging is already perfectly integrated into the infrastructure of the pharmaceutical industry, and it will eventually reduce the costs and time required for novel drug development for lymphoma.

## Figures and Tables

**Table 1:** Characteristics of molecular imaging modalities.

Imaging Modality	Type of Signals	Spatial Resolution	Probes/Needed Amount	Sensitivity (1/(Probe), In Vivo Concentration)	Radiation Hazard	Clinical Translation
Ultrasound	High frequency sound waves	30–500 μm	Microbubbles μg-mg	10^−9^–10^−12^ moles	No	Good
MR	Radio waves	50–250 μm	Gadolinium, iron oxides μg-mg	10^−4^–10^−7^ moles	No	Good
Nuclear (SPECT or PET)	γ rays	0.3–2 mm	Radioisotopes ng	10^−12^ moles	Yes	Good
Optical (Bioluminescence or Fluorescence)	Visible light or near infrared	1–5 mm	d-luciferin, coelentrazine, fluorophore ng-μg	~10^−17^ moles	No	Limited

MR: Magnetic resonance, SPECT: Single-photon emission computed tomography, PET: positron emission tomography.

**Table 2:** Drug discovery and therapy for lymphoma via molecular imaging.

Drugs or Therapy Used	Imaging Modality	Target Receptor/Protein	Lymphoma Type
Rituximab	BLI	CD20	NHL [75]
PS-341 and zoledronic acid (bisphosphonate)	BLI	NA	HTLV-1 infected cell lines [74]
Cyclophosphamide	BLI	NA	Acute lymphoblastic leukemia [78]
Temozolomide	BLI	NA	CNS lymphoma—Raji cells [79]
Lenalidomide plus R-CHOP (phase II trial)	^18^F-FDG PET/CT	NA	DLBCL [104]
^90^Y-ibritumomab tiuxetan and ^90^Y-ibritumomab tiuxetan plus R-CHOP (phase II trial)	^18^F-FDG PET/CT	CD20	NHL, DLBCL [106,107]
JAK2 inhibitor (SB15180) (phase I trial)	^18^F-FDG PET/CT	NA	Relapsed or refractory HL or NHL [105]
^90^Y-daclizumab	^18^F-FDG PET ^111^In-daclizumab SPECT	CD25	Relapsed or refractory HL [110]
^90^Y-rituximab	^89^Zr- rituximab PET/CT	CD20	CD20+ B-cell lymphoma [109]
Tenarad (^131^I-F16SIP) (phase I/II trial)	^18^F-FDG PET/CT	NA	Recurrent HL [108]
¹³¹I-rituximab	^18^F-FDG PET/CT	CD20 with radioimmunotherapy	B-cell non-Hodgkin‘s lymphomas [126]

BLI, bioluminescence imaging; CNS, central nervous system; DLBCL, diffuse large B-cell lymphoma; ^18^F-FDG PET/CT, 2′-deoxy-2′-[fluorine-18]fluoro-d-glucose positron emission computed tomography; HL, Hodgkin’s lymphoma; HTLV, human T-lymphotropic virus; JAK2, Janus kinase; mAb; monoclonal antibody; NHL, non-Hodgkin’s lymphoma; PS-341, proteosome inhibitor; R-CHOP, rituximab, cyclophosphamide, doxorubicin (hydroxydaunomycin), vincristine, Prednisolone; F16SIP, antibody fragment targeting extra-domain A1 of tenascin-C. NA: Not applicable.

## Abbreviations

IND	Investigational new drug
HD	Hodgkin’s disease
NHL	Non-Hodgkin’s lymphoma
MRI	Magnetic resonance imaging
SPECT	Single-photon emission computed tomography
PET	Positron emission tomography
DWI	Diffusion-weighted imaging
FDG	Fluorodeoxyglucose
NIR	Near-infrared
BLI	Bioluminescence imaging
HTS	High-throughput screening
ATLL	Adult T-cell lymphoma/leukemia
HTLV-1	Human T-cell lymphotropic virus type 1
mAb	Monoclonal antibody
ALL	Acute lymphoblastic leukemia
R-CHOP	Rituximab, cyclophosphamide, doxorubicin, vincristine, prednisone
